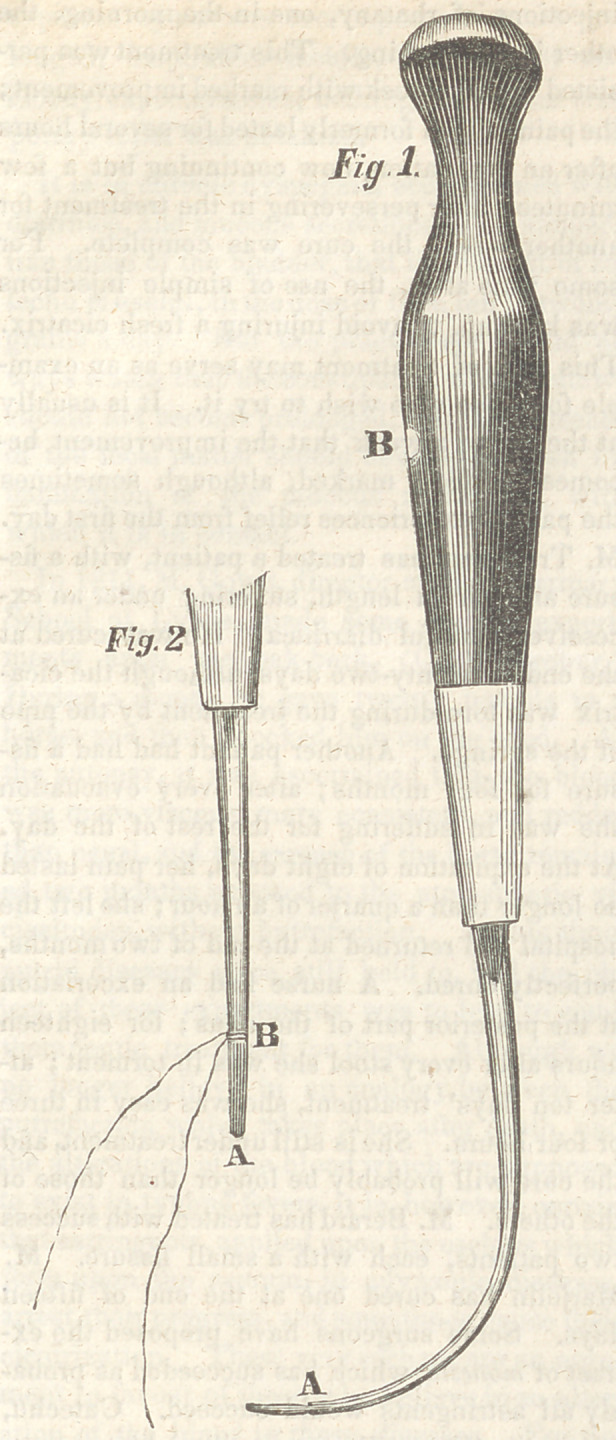# A Case of Amputation at the Shoulder Joint, with a Description of a New Instrument for Securing Deeply Seated Arteries

**Published:** 1840-08-29

**Authors:** W. E. Horner

**Affiliations:** Professor of Anatomy in the University of Pennsylvania


*A Case of Amputation at the Shoulder Joint, with a description of a new instrument for securing deeply seated arteries.* By W. E. Horner, M. D., Professor of Anatomy in the University of Pennsylvania.

The lines of incision for the removal of the upper extremity at the shoulder joint, have been so much varied by different authorities since Le Dran's original operation about the year 1731, that little remains for the surgeons of this day except to select from the numerous routes which have been traced for them. We may consider the modes of operating as reducible to three: first, a circular incision of the integuments, making a flap of them, and a subsequent circular incision of the muscles, the steps being very much as in common amputations. Upon this plan I saw the late Dr. Dorsey, assisted by Dr. Physick, operate in 1816 for a fungus hæmatodes of the arm. The patient being seated on a chair, the subclavian artery was compressed by myself upon the first rib with the fore finger of one hand, sustained by that of the other. The arm being excised, the axillary artery was then secured, and with but little bleeding.* The second mode consists in making a semi-elliptical flap on the anterior, and another on the posterior semi-circumference of the joint, out of integuments and muscles conjointly. Le Dran's, Sharp's, Broomfield's, and Dessault's proceedings, are represented under this. The third plan consists in making the flaps from the deltoid muscle and its integuments, this having been introduced by La Faye, and variously modified in subordinate points by different surgeons, and more especially those of the French schools.

The operation of Dr. Dorsey was performed with the dexterity which distinguished that lamented surgeon, and had a happy termination. The time consumed in it amounted to eleven minutes, including that portion devoted to securing the cut vessels. The axillary artery was evident at the first glance, as he states, and did not bleed; the arrangement for arresting its circulation having been found effectual. The reflections arising from witnessing this case, were, however, that a higher security might be given to the operation by a different position of the patient, and by a different period for securing the artery. For if, upon the moment of excising the limb, the patient from faintiness had fallen from his seat, or any unsteadiness had occurred in the pressure upon the subclavian artery, the chances of a fatal, or at least a very profuse bleeding, were imminent. The conclusion to which I came, therefore, was, that the preferable process belonged to that body of surgeons who adopted the recumbent position, and who, in addition to the advantage from a provision to press the subclavian artery on the first rib, likewise took up the axillary before the severance of the limb.

The facility with which the axillary artery can be reached by adopting a recumbent position, and laying open the front of the axilla in the earliest stages of the operation, would scarcely be anticipated by one who had not witnessed the proceeding; and it affords to this otherwise formidable act of surgery, a security from hæmorrhage certainly not to be exceeded by ordinary inter-articular amputations, where we have the resort of the tourniquet. It may possibly be a point of discussion whether the axillary artery is to be exposed by opening the axilla previously from behind, as recommended by Larrey, or by opening it in front, as exhibited in the following case; the latter proceeding, however, recommends itself by the fact of greater ease to most persons of a dorsal decubitus, and by the certainty of not coming upon the axillary artery before the axilla is fully laid open; a circumstance more likely to happen from the contiguity of this vessel to the latissimus dorsi, where the exposure from behind is resorted to.

Joseph Reynolds, aged thirty-three, of good constitution, a labourer in Mr. Bucks' stone-

---

* See Dorsey's Elements of Surgery, p. 319, vol. 2; Philadelphia, 1818.

quarry, on Ridley creek, near Chester, in the act of withdrawing an instrument, called needle, from a rock, in the process of blasting, produced an explosion of the charge, amounting to about four ounces of powder. The result was an extensive lacerated wound of the left arm, beginning at the under side of it a little below the axilla, and proceeding obliquely upwards. It was probably produced by the aforesaid instrument. The *os humeri* was grazed, but not broken; the upper end of the brachial artery was torn in two, also the brachial vein; all of the adjoining nerves, as the median, ulnar, spiral, and two cutaneous, were laid bare; the coraco brachialis and biceps flexor were cut in two, and the insertion of the pectoralis major was lacerated and exposed. A severe contusion was inflicted on the adjoining lateral part of the chest, and the face was very extensively discoloured by the grains of powder which were driven into the skin; the cornea of the right eye was perforated by some grains of the powder, the left eye was also injured. The ends of the indicator, middle, and ring finger of the left hand were lacerated, the first down to its root; the fingers of the right hand also suffered to some degree by laceration. The quantity of blood lost on the spot was estimated at one and a half gallons. The assistance of Drs. W. Gray, Jos. Porter, and George L. Taylor, living near, was got at an early period, and the wound of the arm secured from further hæmorrhage, for the time, by a thick compress on the axillary artery, and a tight bandage.

The accident occurred at four o'clock, P. M. of Monday, May 25th: the next day, a messenger being despatched for me, I joined the consultation of the above gentlemen about three o'clock, P. M. The patient was then in an extreme state of prostration. The respiration heavy and laborious, with a weak, quick, and frequent pulse; the left arm was much tumefied, pulseless, and with a very distinct crackling of air under the skin; when the limb was stroked from below upwards, the emphysematic air could be forced out in part at the wound. The inferior orifice of the brachial vein under this pressure, discharged black fluid blood mixed with air. Sensibility to a squeeze or puncture existed on the posterior semicircumference of the arm, half way down to the elbow; lower than that, it was equivocal, and on the forearm and hand was decidedly extinguished. The indications were conclusive that the life of the limb below the wound was destroyed, except the posterior part of the arm above the elbow, and that putrefaction had began, which was further indicated by the black and mahogany colour of the integuments. Having removed the bandage and compress, the wound exhibited itself as described. I found, without difficulty, the lacerated orifice of the brachial artery, which was plugged with a firm coagulum of blood; over it I threw a ligature and then did the same for the vein, so as to prevent the recurrence of hæmorrhage, while a careful search was made into the extent and character of the injury.

A consultation with the above gentlemen was then held, whether, under the circumstances, it was proper to perform the excision of the limb at the joint, the prospect of final recovery being so gloomy, and even of the patient having strength to go through the operation. The latter was finally concluded on, the consent of the wife and friends sanctioning it, upon a candid representation of its unpromising results.

The following process was adopted to prevent further exhaustion of the patient. He was permitted to remain in bed and on his back, the shoulders being elevated, and he drawn near the left side of the bed. A semi-elliptical flap was made in front of the joint extending from the point of the acromion to the centre of the axilla, it being formed from the integuments, deltoid, and great pectoral muscle. This exposed fully the axillary nerves and blood vessels. A ligature was then passed around the axillary artery and another around the axillary vein with my hæmostatic needle, which I found on this, as on several former trying occasions, a most convenient instrument for securing large and deeply seated blood-vessels. The danger from hæmorrhage thus obviated, I proceeded to make on the posterior face of the shoulder a semi-elliptical flap, out of the posterior half of the deltoid, the latissimus dorsi, and teres major,—it extending from the point of the acromion to the centre of the axilla also. The axillary nerves and blood-vessels were cut through previous to making the posterior flap. I now proceeded to the disarticulation of the joint, which was easily accomplished. The whole of the above incisions, with the exception of the division of the posterior part of the deltoid, was executed with a scalpel; and the time consumed was inconsiderable, except that devoted to the securing of blood-vessels, large and small. Not two ounces of blood were lost from the operation, and the patient bore it well. The wound, after being exposed a short time to the air, for the purpose of affording an opportunity to any small vessels to bleed which had previously escaped detection, was then closed by the approximation of the flaps; the latter were secured by a stitch in the centre, and strips of sticking plaster, and over the latter was placed a thick compress, secured by a bandage around the thorax.

Having left the patient about sunset to return to the city, I learned through a communication from Dr. Gray, a few days afterwards, that he continued to decline in strength, and died about nine o'clock on the evening of the operation.

The needle used is represented in the following sketch:

The points of principal convenience in this instrument are, 1st, the shape of the handle, Fig. 1, which is formed of ebony, with a silver ferule, and a mark at B to indicate, when the needle itself is concealed, the direction of its concavity; 2d, the line of curvature of the needle, and the shape of its end, which is not pointed, but rounded and cutting, as in Fig. 2, A; and, 3d, the ligature, being attached not by being passed through an eye, but by being thrown around the needle by what is called, in nautical phraseology, a clove-hitch, as in Fig. 2, B. A small notch at A, Fig. 1, is the spot to which the ligature is thus fastened.

The needle is fixed permanently in the handle, but it may be so arranged as to admit of removal; and I have, accordingly, instruments thus constructed.

It is a matter of common experience in surgery, that a needle fixed firmly in a handle has many recommendations in its application to large and deep seated arteries, from the action of the point being much more easily regulated; hence the inventions on that ground are very numerous. Where, however, this arrangement is adopted, the eye must be near the point of the needle, so as to convey the ligature under the artery; but in placing the eye there, the needle is too much weakened, unless it be made inconveniently large. It was with the view to meet this objection, that I have devised as a novelty in my own instrument,* a notch like a shoulder, which detracts but little from its strength, but which is quite sufficient to prevent the clove-hitch of the ligature from slipping backwards, while it may be very readily slipped forwards, so as to detach it at the pleasure of the surgeon. The ligature is also more readily attached by the clove-hitch than it could be passed through an eye, which is all-important in repeated and hurried applications. The flattened cutting semi-circular or rounded point is also a decided advantage over the pricking point, as it turns off more readily from the cylindrical surface of the artery, and less hazard is run of puncturing the latter.

This instrument I have, by experience of its use in some urgent cases, found to be well adapted to the subclavian, external iliac, and femoral arteries; in fact, it is so to all that are deep-seated, and within reach.

* For the diversity of instruments used in taking up arteries, see Seerig Armamentarium Chirurgicum, Breslau, 1838; it being a very elaborate historical and graphic illustration of surgical instruments of all kinds and dates.

## Figures and Tables

**Fig. 1. Fig. 2 f1:**